# Personal and Network Dynamics in Performance of Knowledge Workers: A Study of Australian Breast Radiologists

**DOI:** 10.1371/journal.pone.0150186

**Published:** 2016-02-26

**Authors:** Seyedamir Tavakoli Taba, Liaquat Hossain, Robert Heard, Patrick Brennan, Warwick Lee, Sarah Lewis

**Affiliations:** 1 Complex Systems Research Group, Faculty of Engineering & IT, the University of Sydney, Sydney, New South Wales, Australia; 2 Division of Information & Technology Studies, Faculty of Education, the University of Hong Kong, Pokfulam, Hong Kong; 3 Health Systems and Global Populations Research Group, Faculty of Health Sciences, the University of Sydney, Sydney, New South Wales, Australia; 4 Medical Image Optimisation and Perception Group (MIOPeG), Faculty of Health Sciences, the University of Sydney, Sydney, New South Wales, Australia; Semmelweis University, HUNGARY

## Abstract

**Materials and Methods:**

In this paper, we propose a theoretical model based upon previous studies about personal and social network dynamics of job performance. We provide empirical support for this model using real-world data within the context of the Australian radiology profession. An examination of radiologists’ professional network topology through structural-positional and relational dimensions and radiologists’ personal characteristics in terms of knowledge, experience and self-esteem is provided. Thirty one breast imaging radiologists completed a purpose designed questionnaire regarding their network characteristics and personal attributes. These radiologists also independently read a test set of 60 mammographic cases: 20 cases with cancer and 40 normal cases. A Jackknife free response operating characteristic (JAFROC) method was used to measure the performance of the radiologists’ in detecting breast cancers.

**Results:**

Correlational analyses showed that reader performance was positively correlated with the social network variables of degree centrality and effective size, but negatively correlated with constraint and hierarchy. For personal characteristics, the number of mammograms read per year and self-esteem (self-evaluation) positively correlated with reader performance. Hierarchical multiple regression analysis indicated that the combination of number of mammograms read per year and network’s effective size, hierarchy and tie strength was the best fitting model, explaining 63.4% of the variance in reader performance. The results from this study indicate the positive relationship between reading high volumes of cases by radiologists and expertise development, but also strongly emphasise the association between effective social/professional interactions and informal knowledge sharing with high performance.

## Introduction

Job performance is a central focus for researchers and policy makers from diverse domains. Different studies have tried to provide an appropriate understanding of performance constructs, particularly in knowledge intensive work, which requires a workforce with the ability to conduct complex analytical tasks within their daily role. In this regard, two main groups of studies can be considered: studies which focus on individual characteristics, behaviours and attitudes, and studies which emphasise social network influence, information flow and knowledge/expertise sharing. The nature of our research is to consider both approaches simultaneously in order to examine the performance of knowledge intensive workers.

Radiologists are a very good case of knowledge intensive workers because their work largely consists of the application of knowledge in correct image interpretation, also known as “observer performance” in the literature. Choosing breast radiology as the domain of this study provided the researchers with the ability to quantify medical performance through objective measures, as opposed to many previous network studies that measured performance through subjective proxies such as perception [1]. Earlier studies on good observer performance in mammography, the primary modality of breast imaging, show an association to readers’ personal characteristics, particularly the readers’ experience and case load. In these studies, experience factors such as years reading mammograms, number of mammograms read per year and hours reading mammogram per week are positively correlated with performance [2–5]. This paper extends this traditional understanding of performance in breast radiology to a level which also includes significant effects of social networks. Social/professional networks are defined in this study as consisting of professional people with whom radiologists associate, interact or work in their occupation.

## Theoretical Underpinnings

Bernardin and Beatty [6] define performance as the recorded output or results of a particular job activity in a particular time frame. Many researchers have tried to explore which personal characteristics have a direct bearing on job performance. A study by Hunter [7] shows that job knowledge and skill influence job performance. Similarly, McCrae and Costa [8] present a meta-theoretical framework, which indicates there are associations between qualities such as personality, knowledge and skill, and performance. Moreover, Campbell et al. [9], and later Campbell et al. [10], developed a theory of performance, which suggests that declarative knowledge, procedural knowledge/skill and motivation are the three direct dynamics of job quality and performance.

Declarative knowledge is the factual information, rationales and actions which are perceived by someone and can be evaluated and quantified, such as through examination papers. Task-related information and declarative knowledge have a positive effect on performance [11]. Procedural knowledge, on the other hand, refers to the knowledge about how to do a certain work task, the skill to follow procedures at work and the capability to carry work out effectively. In this regard, it is hands-on real work experience, which augments procedural knowledge [12] and finally leads to better performance. Feedback [13], and particularly social feedback [14], has a critical role in this knowledge development process. Moreover, previous research shows that personal qualities and interests interact with qualifications, training and experience to form both declarative knowledge and procedural knowledge, which lead to improved performance [15]. Particularly, Pierce et al. [16] and Gardner and Pierce [17] show that self-esteem is positively associated with the job performance of individuals. From the above discussions, it can be asserted that three personal characteristics would be positively associated with job performance in knowledge intensive works: declarative knowledge, work experience and self-esteem/self-evaluation.

Social networks literature suggests the inherent role of different social network structures and characteristics in facilitating or hindering knowledge transfer, information exchange, feedback processes and improved performance. In a professional/collegial network setting, individuals are actors, and their professional relations with other contacts are ties/links. Previous research reveals that, all things being equal, individuals with a higher number of contacts (degree centrality) tend to show greater levels of performance [1,18–20]. However, Burt [21] suggests that the maintenance of social connections could be costly and time consuming. Redundancy (receiving similar information and benefits) occurs when a person communicates with other contacts who are directly connected with each other or indirectly connected through shared contacts. In view of that, he developed the concept and measure of networks’ effective size to indicate the number of non-redundant contacts of an actor. In his structural holes theory, Burt [21] addressed the benefits gained to members of a network who serve as a channel of information transfer among otherwise unrelated contacts and clusters in the network and so have effective and efficient relations. Accordingly, it can be stated that greater network degree centrality, effective size and efficiency (simply the ratio of effective size to degree centrality) should have a positive relation with performance.

Conversely, Burt [21] defined network constraint as a measure of the extent to which an actor’s time and energy are invested in contacts who are themselves connected to one another. Burt [20] considers constraint as the best summary measure of benefits gained by an actor in the network (with a negative relationship between constraint measure and actual benefits). Network constraint varies with three dimensions: network size, network density, and network hierarchy. In general, actors with larger network size receive more diverse information and so are less constrained. However, network density, which determines the average connectedness amongst actors, leads to constraint because it increases the likelihood of sharing the same information via different contacts. Hierarchy is also a measure which shows unequally distributed network constraint imposed by a limited number of contacts and has been showed to be positively related to constraint [20]. It is postulated that greater network constraint, density and hierarchy are all negatively related to performance.

The diversity and extensiveness of an actor’s personal connections may also impart a significant effect upon originality and credibility of information he/she receives. In this regard, Cross and Cummings [19] suggest that in knowledge intensive work, actors who span ties to diverse occupational levels or physical/geographical places have higher performance. Thus, it is expected that geographical and functional diversity would be positively associated with performance.

Besides the exploration of positional and structural effects of the network, researchers have also considered the influence of network relational properties on the flow of information and knowledge. At a relational level, the quality of tie/link between two actors is considered as the tie strength. Emotional closeness (intensity) of two actors, frequency of their interactions and duration of their connectedness are the most important properties of their relation in terms of tie strength [22,23]. In investigating social relations of knowledge intensive workers, recent studies argue that strong ties are more efficient in transferring complex information among actors and are so more beneficial [1,24,25]. Accordingly, in this study, it is postulated that greater network tie strength is positively related to performance.

**Fig 1:**
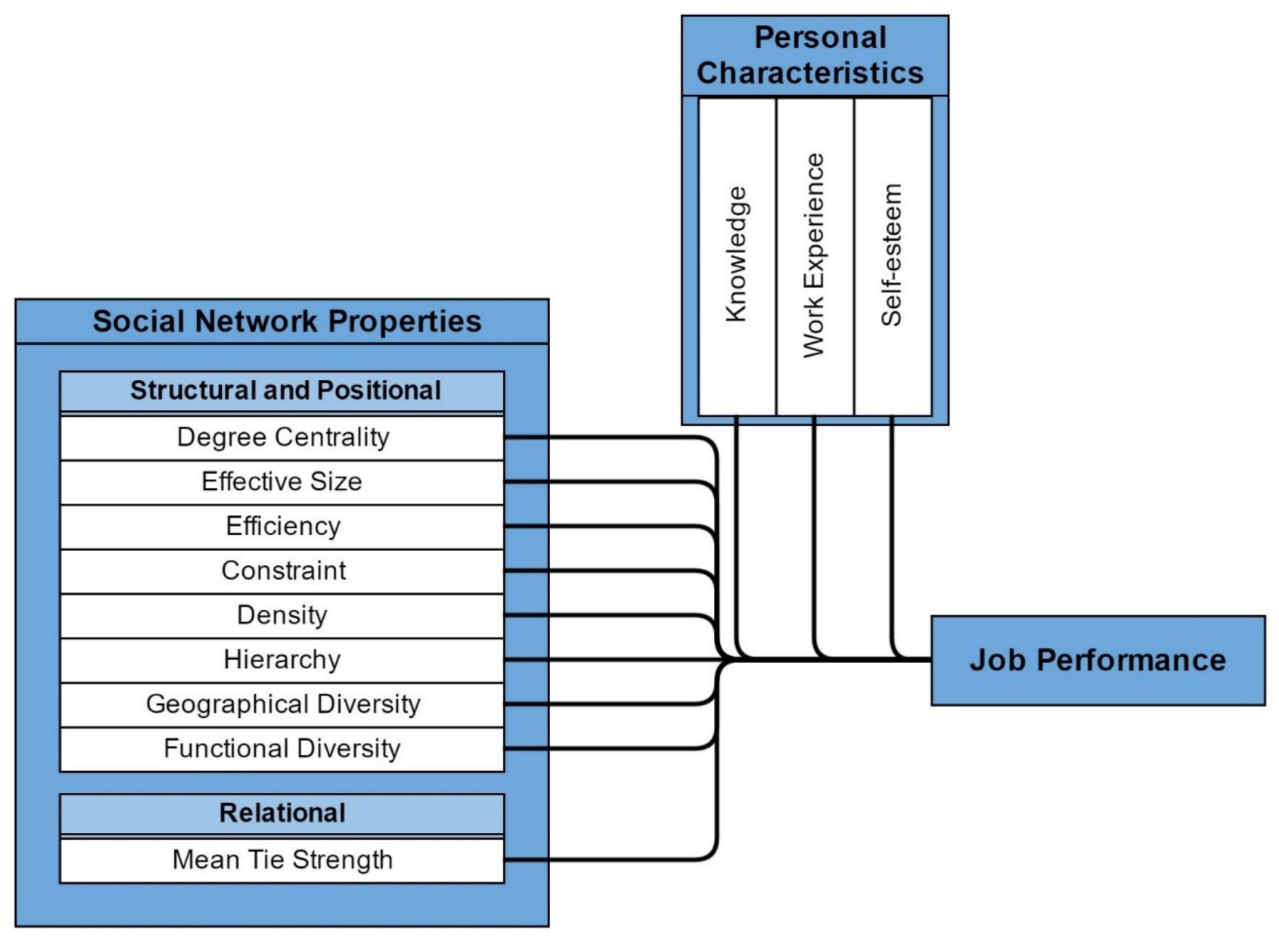
Theoretical model for job performance constructs in knowledge intensive works.

Based upon the above discussions, a theoretical model, Fig 1, can be considered for studying the relationship between social network properties, personal characteristics and job performance of knowledge intensive workers.

## Methods

With the objective of examining the performance constructs of Australian breast imaging radiologists, we designed a mixed methods research study. This study was comprised of, firstly, a data collection of professional networks and attributes of radiologists through a survey instrument and, secondly, an observer performance test through the BreastScreen Reader Assessment Strategy (BREAST). BREAST is a radiology training and quality assurance tool that simulates the clinical reading tasks of radiologists who read mammograms for the national breast cancer screening program, BreastScreen Australia, and provides educational feedback about performance.

For collecting and analysing data about social/professional networks, there are two different approaches; the sociocentric (full network) approach, which examines social structural and social relational patterns in the whole network [26] and the egocentric (personal networks) approach, which centres on each sample focal node (ego) and his/her directly related contacts (alters) in order to understand the social attributes that linked to the ego [27]. We define ego in network parlance to mean “the person being investigated”, and alters as “the people who are the ego’s affiliates or the others to whom the ego is linked” [28]. In this study, we used an egocentric approach for data collection, where each person (ego) was asked to describe his/her directly related contacts (alters). A sociocentric approach was not attempted in this preliminary study, due to the logistical and ethical complications inherent in such data collection. We sent an invitation to participate in the survey study to all 153 breast imaging radiologists on the BREAST contact list around Australia, and 31 (20%) completed the purpose designed questionnaire regarding their network characteristics and personal attributes. These radiologists also independently reviewed a test set of 60 mammographic cases (240 mammographic images in total, 4 image per case) using the BREAST platform in order to assess their performance. Ethics approval was received from the University of Sydney Human Research Ethics Committee (Project No.: 2014/485) and participants provided their written consent to participate in this study.

### Survey Instrument

An egocentric approach has been considered a reliable and practical method for data collection in previous social network studies [29–33]. We constructed our study upon the Burt [34] argument for obtaining network data in the General Social Survey (GSS) and the methodology described by West et al. [30], Knoke and Yang [32], Chung and Hossain [1] and Burt et al. [35].

**Fig 2:**
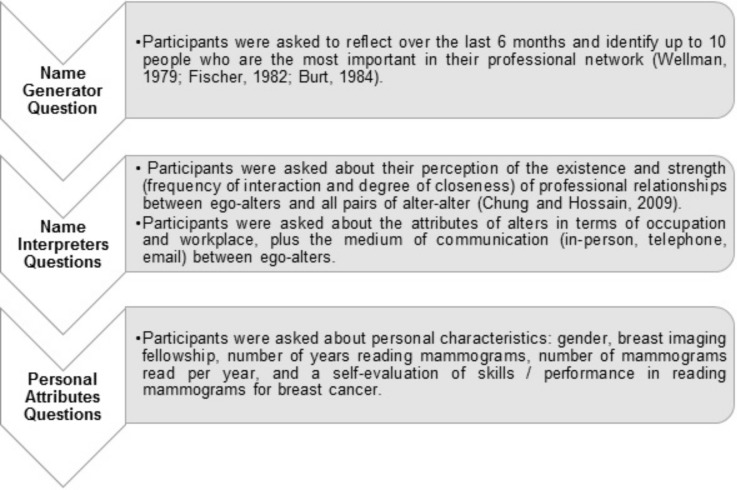
Schematic flow of three levels of questions in the social network survey.

Radiologists were provided with a definition of a “social/professional network” as consisting of professional people with whom they associate, interact or work in the radiology domain. Based upon the Burt et al. [35] study, we explained to radiologists that they may interact with their network for different reasons such as discussing important matters with other trusty people, having informal social activities (for example lunch, coffee), helping to advance professionally over the course of their professional career, discussing difficult cases in their practice, talking about routine clinical/operational matters and considering quality improvement in procedure and patient care. We facilitated an online tool for managing three sets of questions in our questionnaire. Fig 2 shows a schematic of different layers of the survey tool.

### Observer Performance

The participating radiologists gave consent to access their observer performance data in interpreting mammograms from the BREAST database. The methodology of the interactive BREAST mammographic screen reading test set for measuring the observer performance was as follows: each radiologist read a test set of 60 mammographic cases obtained from BreastScreen New South Wales. Twenty of the 60 cases were abnormal and the remaining 40 cases normal. Abnormal cases contained biopsy-proven single malignant lesion. Cases classified as normal were confirmed by a two year negative re-screen.

**Fig 3:**
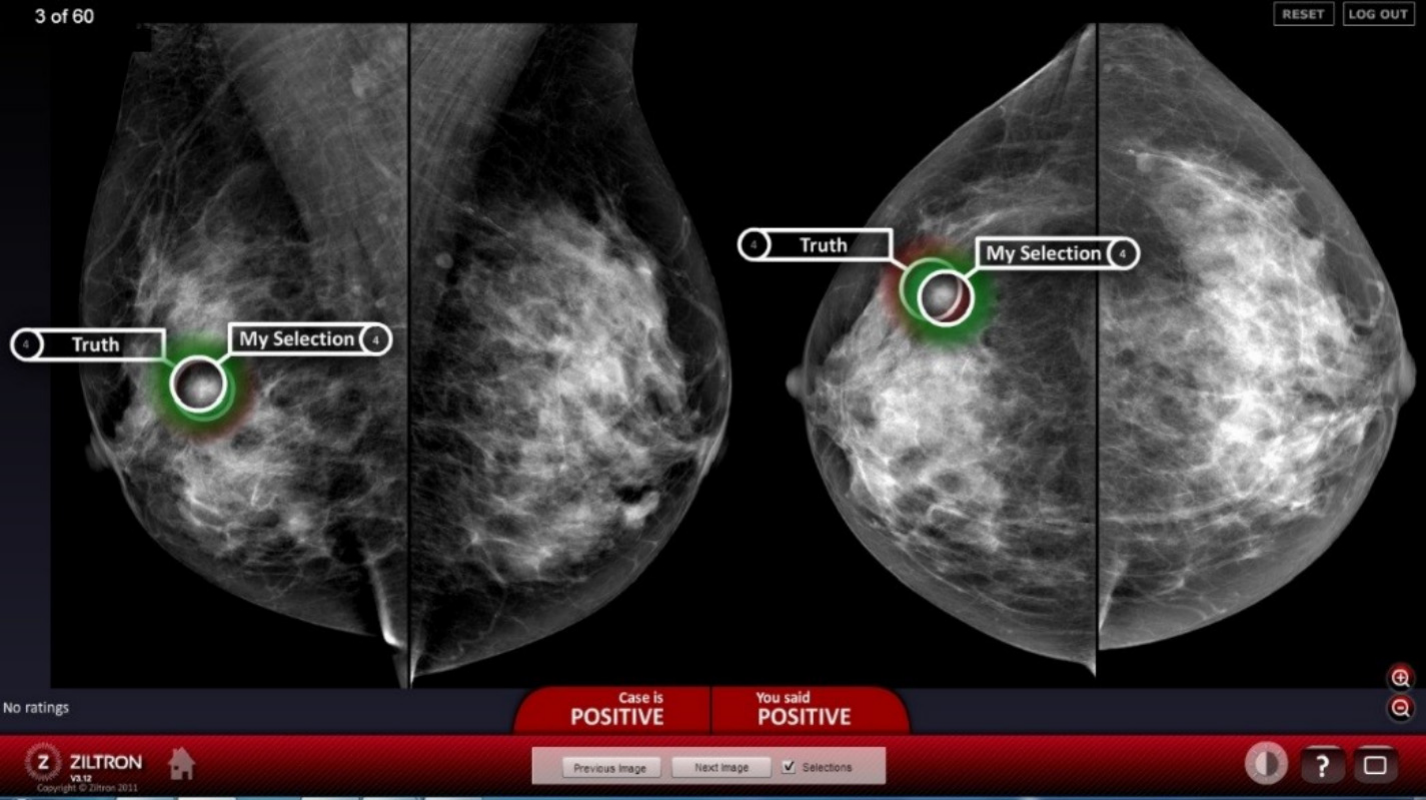
A screenshot of the BREAST tutorial evaluation for measuring the observer performance.

For each abnormal case, the radiologists were asked to identify the location of any malignant lesions, specifying their level of confidence on a scale of 1 (completely confident that the case was normal) to 5 (completely confident that the case was malignant). For the purpose of the performance test, radiologists had access to post-processing tools such as windowing the contrast and brightness of the images and magnification. The radiologists were free to advance forwards and backwards through the cases and correct their answers if desired. The reading environments were matched to standard radiological reporting environments in that all cases were displayed on industry approved equipment including two 5 MP monitors driven by a Sectra key-pad system, which is a familiar reading set up in BreastScreen Australia. At the conclusion of the test set, the radiologists were presented with feedback about their performance. Fig 3 shows a screenshot of one BREAST tutorial test and the feedback received by the radiologist.

The BREAST database provides measurements on radiologists’ sensitivity, specificity, location sensitivity and Jackknife free-response operating characteristic figure of merit (JAFROC FOM) values. The JAFROC FOM was chosen as the radiologists’ primary performance variable as it describes both the ability of radiologists to locate malignant lesions (their location sensitivity) and probability that a confidence rating applied to a correct lesion (a true positive decision) exceeds a confidence rating applied to a false-positive decision on a normal case. JAFROC FOM is accepted in radiology research as the highest measure of performance [36].

### Data Analysis

Data from the survey instrument was organised by Microsoft Excel and the social network data was analysed using E-NET (version 0.41) [37], a program that specialises in the analysis of egocentric networks. We used the E-I (external-internal) statistic [38] to investigate the similarity of characteristics (Homophily) between each ego and his/her alters in terms of workplace, occupation and gender. The E-I statistic shows the network connectedness between various groups by subtracting the number of out-group ties from the number of in-group ties and dividing this difference by the total number of ties. The E-I statistic varied between -1 for radiologists who only interacted with alters in the same group (for example alters from exclusively the same workplace), and +1 for those who only interacted with alters in other groups (for example alters who were exclusively from another occupational role, i.e. non-radiologists). The diversity of alters was also worked out using Teachman's Entropy Index [39,40] in order to indicate the extent to which they belong to groups/categories that are different.

Observer performance and all social network parameters were measured and analysed as continuous variables. We used weighted ties (average of frequency and closeness) to calculate the strength of network ties. Personal characteristics were measured as ordinal variables (except for gender and having fellowship training, which were nominal) and then dichotomised (median as the cut-off point) for correlation and regression analysis. SPSS software (version 22.0) was used for all statistical calculations. In order to test the theoretical model developed in the previous section, we correlated BREAST performance score (JAFROC FOM) against personal characteristics and network properties. The 95% confidence intervals of each predictor were also calculated based upon 1000 bootstraps.

Additionally, a two-step hierarchical multiple regression was conducted with JAFROC FOM as the dependent variable, where personal characteristics predictors were entered at the first step and social network parameters entered at the second step. SPSS was programmed to produce the total R squared value after each step, and also the increase in R squared when the social network predictors were added to the model. Initially, all predictors which had a tendency to be significant in correlation tests (P ≤.10) were considered for the regression analysis, but then one (constraint) which caused multi-collinearity was excluded. From the remaining six predictors, those two without unique significant contribution to the model (P ≥.05) were also omitted in the final model. We also checked for univariate outliers, multivariate outliers, normality, linearity and homoscedasticity, and all the assumptions were satisfied. Multiple regression R^2^ values were tested for significance by using F ratios.

## Results and Discussions

### Statistics

From the total of 31 participants, 13 (41.9%) were male and 18 (58.1%) were female. The JAFROC FOM varied between 0.56 and 0.91 (M = 0.77, SD = 0.09), which provided a good spectrum for further analysis of performance constructs. The participants were a highly experienced sample of Australian radiologists. The majority (n = 18, 58.1%) of participants had high (more than 15 years) experience in reading mammograms and 45.2% (n = 14) had a very high volume of (more than 10,000) mammograms read per year. Also, 29% (n = 9) of radiologists had completed a fellowship in breast imaging. In self-evaluating their observer performance, 80.6% (n = 25) of radiologists evaluated themselves as above the competent (good and expert) in reading mammograms.

The number of alters (equal to degree centrality) in the egocentric networks of radiologists varied between 2 and 10 (M = 8.06, SD = 2.33). In accumulating data on 249 alters who were nominated by participants, females (54.6%) had a slightly larger ratio than males (45.4%). The majority (57.0%) of alters in breast radiologists’ networks were other radiologists, followed by radiographers (9.6%) and surgeons (9.2%). It was found that 94.8% of alters were from exactly the same place or the same city as the ego’s workplace, which reflects the intensely centred knowledge sharing network of radiology. On average, 58.2% of communications between ego-alters were exclusively in-person and without extended information and communication technologies (ICT). Table 1 shows some important attributes about alters in detail.

**Table 1:** Detailed demographic information about alters in egocentric networks of breast radiologists.

*Characteristics of Alters*		*Number of alters*	*Percentage of alters*
Gender	Male	113	45.4%
	Female	136	54.6%
Occupation	Radiologist	142	57.0%
	Surgeon	23	9.2%
	Pathologist	7	2.8%
	GP or Resident Doctor	2	0.8%
	Clinician (Tertiary Specialist)	12	4.8%
	Radiographer	24	9.6%
	Nurse	1	0.4%
	Health Administrator/Manager	20	8.0%
	Other	18	7.2%
Workplace	Same place as ego's	188	75.5%
	Different place in ego's city/town	48	19.3%
	Different city/town from ego's	10	4.0%
	Different state from ego's	2	0.8%
	Different country from ego's	1	0.4%
Medium of communication (ego- alter)	In-person	145	58.2%
	Telephone	27	10.8%
	Email	14	5.6%
	Video conference	0	0.0%
	Others	20	8.0%
	Combination of all	43	17.3%

Calculating E-I scores at ego-level and population-level, the results indicate that radiologists have a tendency towards sharing ties with alters in the same group or class as self. The population-level E-I for workplace was—0.51, which is considered a medium/high propensity. More interestingly, 38.7% of radiologists did not have any professional interaction with others out of their own workplace (E-I score of -1). We also found a small preference for radiologists to network with alters of the same gender (-0.26) and the same occupation (-0.14).

### Correlational Analyses

The correlational analyses of BREAST performance score against personal characteristics and network properties are presented in Tables 2 and 3, respectively. In our dataset, JAFROC FOM had a weak/moderate positive relationship (r = 0.39, P = .029) with experience in terms of reading 10,000 or more mammograms annually but a non-significant positive correlation with number of years’ experience in reading mammograms. The positive correlation between performance and having a fellowship in breast imaging, as a measure of specific knowledge/qualification in breast imaging, was also not significant; however less than one third of participants in this study had a fellowship training in mammography and this affects the statistical power. In considering radiologists’ attitude to self-performance, those who had a higher self-evaluation (above the competent) did in fact tend to perform better at the BREAST task (r = 0.44, P = .013).

**Table 2:** Point-biserial correlations of (dichotomous) personal characteristic parameters with JAFROC FOM.

*Personal Characteristics Parameter*	*JAFROC*			
	r	P	BCa 95% Confidence Interval	
			Lower	Upper
High No. of mammograms read per year	.393*	.029	.025	.690
High No. of years read mammograms	.250	.175	-.102	.634
Having fellowship in breast imaging	.103	.582	-.171	.331
High self-evaluation	.441*	.013	.145	.751

* P≤.05.

Bootstrap results are based on 1000 bootstrap samples.

**Table 3:** Pearson correlations of social network parameters with JAFROC FOM.

*Social Network Parameter*	*JAFROC*			
	R	P	BCa 95% Confidence Interval	
			Lower	Upper
Degree centrality	.470^**^	.008	.188	.681
Density	.025	.892	-.280	.313
Effective size	.360^*^	.046	.098	.564
Efficiency	-.010	.959	-.421	.395
Constraint	-.410^*^	.022	-.628	-.259
Hierarchy	-.442^*^	.013	-.737	-.074
Mean Tie strength	.304	.097	-.087	.583
Occupational diversity	.155	.406	-.254	.525
Geographical diversity	-.071	.704	-.406	.236

** P≤.001

* P≤.05.

Bootstrap results are based on 1000 bootstrap samples.

The relationship between JAFROC and different social network parameters varied with regard to magnitude, direction and significance. Having more contacts/alters in the egocentric networks was positively related to higher performance of radiologists at a moderate level (r = 0.47, P = .008). Having more non-redundant contacts, measured by effective size, was also weakly/moderately correlated with performance (r = 0.36, P = .046); however, that was not the case for efficiency, probably because degree centrality and effective size counteract the effects of each other in the measurement.

Constraint had a moderate negative relationship with performance (r = -0.41, P = .022). Examining the relationship between constraint and its theoretical constructs, namely network’s effective size, density and hierarchy, it appears that the constraint in egocentric networks of breast imaging radiologists is influenced by effective size and density but not hierarchy. Constraint had a strong negative correlation with effective size (r = -0.75, P ≤.001), a trend towards significant and positive correlation with density (r = 0.336, P = .064), and no relation with hierarchy. Our results also show that hierarchy was negatively correlated with JAFROC FOM at a moderate-level (r = -0.44, P = .013) but density was not. Overall, these results suggest that radiologists who interacted with a closely bonded cluster through multiple primary ties, which resulted in higher constraints for them performed worse than radiologists with effective, less constrained (or non-redundant) contacts.

**Fig 4:**
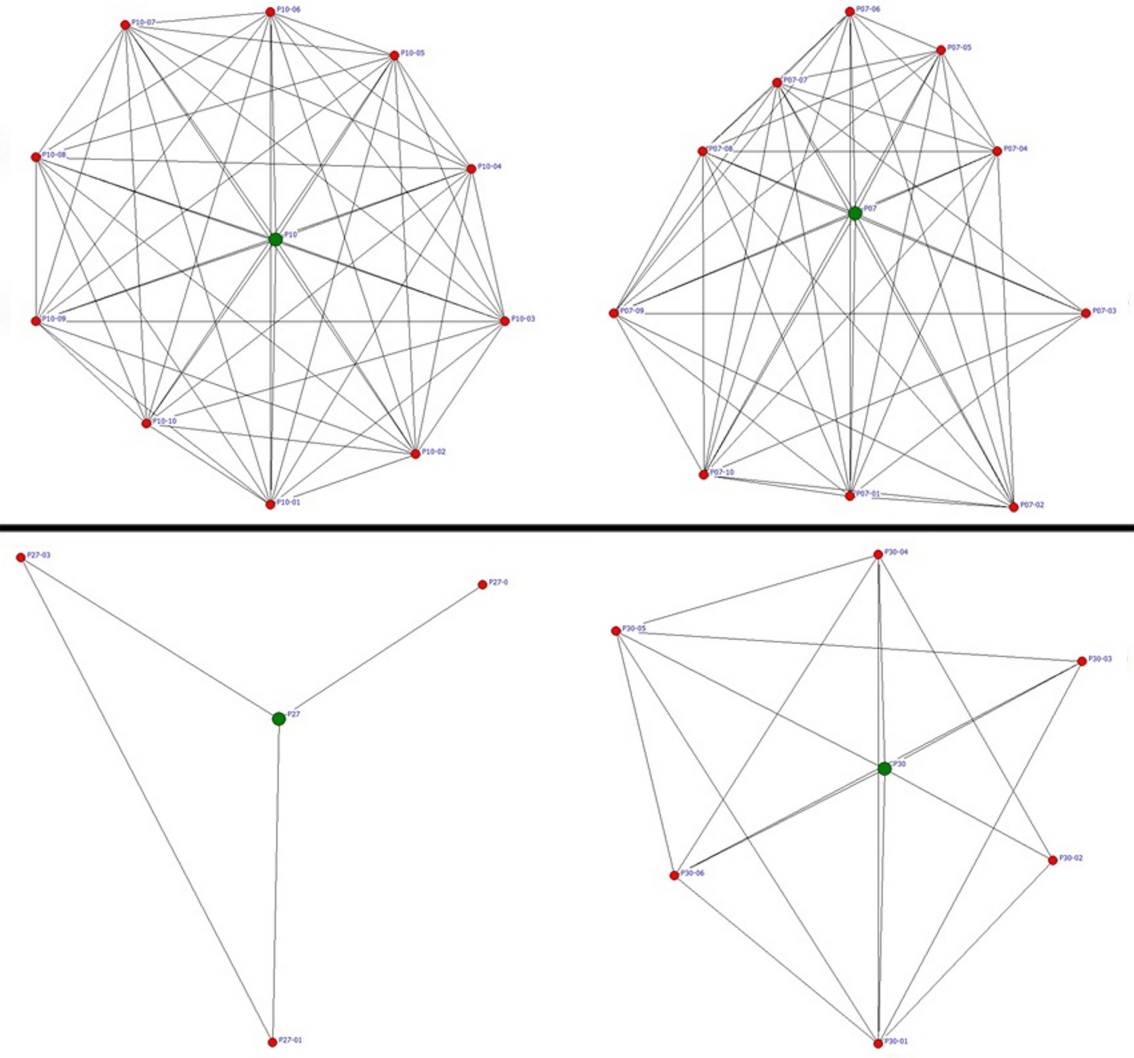
Egocentric professional network of four different radiologists. Upper Row: radiologists with high degree centrality, relatively high effective size, low constraint, low hierarchy and high performance (JAFROC FOM = .91 (upper left) and .91 (upper right)). Lower Row: radiologists with low degree centrality, low effective size, high constraint, high hierarchy and low performance (JAFROC FOM = .56 (lower left) and .59 (lower right)).

Examining the strength of ties in radiologists’ egocentric networks, the mean tie strength of ego had a non-significant positive relationship with performance (r = 0.304, P = .097). The diversity of alters with regard to their occupation and workplace was analysed using Teachman's Entropy Index; however, the results show that diversity did not have any significant effect on performance of radiologists. Fig 4 compares the professional network of four different radiologists, where the upper row shows two examples of radiologists with comparatively high performance in detecting breast cancers and the lower row shows two examples of radiologists with comparatively low performance.

### Hierarchical Multiple Regression

Table 4 displays the different steps of hierarchical multiple regression analysis. The annual volume of mammograms read is considered the most important predictor of JAFROC in the breast radiology literature [5] and thus this predictor was entered at step one. The results revealed that a high number of mammograms read per year accounted for 15.5% of the variation in JAFROC, F (1,29) = 5.305, P ≤.05. Introducing the effective size, hierarchy and tie strength at step two added a substantial contribution to the regression model, F (3,26) = 11.369, P ≤.001, explaining an additional 48.0% of variation in JAFROC FOM. Overall, the combination of all four independent predictors of high number of mammograms read per year, effective size, hierarchy and mean tie strength explained a significant amount of the variance in JAFROC FOM (R^2^ = .634, F (4,26) = 11.276, P ≤.001).

**Table 4:** Hierarchical multiple regression analysis with JAFROC FOM as the dependant variable.

*Model*	*Predictor*	*ΔR*^2^	*b*	*Std*. *Error*	*β*	*P*
Step 1		.155				
	Constant		.740	.020		≤.001
	High No. of mammograms read per year		.068	.029	.393^*^	.029
Step 2		.480				
	Constant		.579	.061		≤.001
	High No. of mammograms read per year		.067	.021	.386^*^	.004
	Effective size		.025	.006	.521^**^	≤.001
	Hierarchy		-1.440	.348	-.498^**^	≤.001
	Mean Tie strength		.034	.017	.246^a^	.053

** P≤.001

* P≤.05

^a^ marginally significant at the 0.05 level.

R^2^ = .634.

## Conclusion

In order to examine the performance of knowledge intensive workers, we developed a theoretical model about personal and network dynamics of job performance based upon the literature and tested the model in the Australian breast radiology context. The results from this study suggest a strong association between social networks and observer performance by radiologists. Network factors accounted for 48% of variance in observer performance, in comparison to 15.5% for the personal characteristics for this study group. The number of contacts and the number of non-redundant (effective) contacts were both positively related to observer performance but the latter was revealed to also have a unique influence on performance when examined simultaneously with all other predictors. Constraint and hierarchy in the professional network of radiologists were also negatively related to their observer performance. In addition, results indicate that strong ties, in terms of frequency of interaction and degree of closeness, between an actor and his/her contacts were advantageous for transferring complex analytical knowledge, such as interpretation of medical images. We believe this is the first time that network factors have been calculated and found to be quantitatively more important than the traditional constructs of work volume/task experience by radiologists, however we exercise caution in acknowledging the unstudied effects between individual training factors and social network parameters.

In terms of personal constructs, the amount of task experience (number of mammograms read per year) was an appropriate predictor of performance and this finding is in agreement with previous research in breast radiology [41]. Previous meta-data analysis of experience research also shows that among different possible sets of measurement methods (amount, type and time) and range of specificity (task, job, organisational) for defining the features of experience, the amount of task has the strongest relationship with quantities of job performance [12]. According to our results, self-esteem was also positively related to performance, but it did not provide a unique contribution to performance. One can also argue that it is indeed performance which influences the attitude and self-evaluation not vice versa.

We acknowledge that there were some limitations in this study. Firstly, similar to all egocentric networks studies, we analysed personal networks of radiologists based upon self-reporting of participants about their contacts and relations. Although this approach is commonly accepted by many social networks analysts, it is less reliable than the sociocentric network approach, which examines social structural and relational properties of the full network. In addition, it has been argued by some researchers that the behaviour of the full network may be beyond the activities of individual actors and so partly a consequence of the local egocentric behaviours [42]. Nevertheless, the fact that the sociocentric approach is only applicable to closed networks with easily determined actors within predefined boundaries, makes this approach impractical for data collection in many real-world case studies.

Secondly, the number of participants in this study was small. This is due to the relatively small workforce of breast radiologists in Australia, their limited availability being highly skilled professionals, and the nature of this research which required radiologists to participate in two different data collection processes for network properties and observer performance. Hierarchical multiple regression analysis with a relatively small n may not accurately estimate the true importance of predictors. Our study included highly experienced radiologists and the inclusion of less experienced participants in future studies may show a different association between parameters.

Thirdly, studying causality was not part of the design of this study. The results from the current study do not show the direction of relationship between specific network characteristics and performance of radiologists. There may be also underlying personality characteristics that cause specific networking behaviour among radiologists. Future studies should consider these possibilities.

In summary, our study of 31 Australian breast radiologists suggests that network factors account for 48% of the variance in observer performance, in comparison to 15.5% for number of cases read per year. The results suggest a strong new direction for research into improving observer performance. Future studies in observer performance should consider social networks influence as part of their research paradigm, with equal or greater vigour than traditional constructs of reading volumes or reading experience.

Breast radiologists working in screening mammography are required to make independent, blinded decisions regarding the presence of cancers. Furthermore, breast radiology training and work environments often mean radiologists may not have direct patient contact, may be physically isolated from other clinicians and work in isolation from their peers [43–45]. The current results raise the possibility that encouraging them to search for further collaborations, exchanges and conversations with other professionals may be an effective means of facilitating expertise development in breast radiology.

The current work provides an important insight for medical educators and policy makers in health. The results from this study emphasise the importance of further investigation of knowledge sharing and social learning through effective professional interactions, in research into improving the skill and performance of the current and next generation workforce. Clinical quality assurance activities such as multi-disciplinary team (MDT) meetings [44] and clinical education review meetings are important events where clinicians can meet and discuss the diagnosis and management of patient cases and may provide suitable settings for more detailed research into the relationship between network characteristics and clinical performance.

## References

[1] Chung KSK, Hossain L (2009) Measuring performance of knowledge-intensive workgroups through social networks. Project Management Journal 40: 34–58.

[2] Miglioretti DL, Smith-Bindman R, Abraham L, Brenner RJ, Carney PA, Bowles EJA, et al (2007) Radiologist Characteristics Associated With Interpretive Performance of Diagnostic Mammography. Journal of the National Cancer Institute 99: 1854–1863. 18073379 10.1093/jnci/djm238 PMC3144707

[3] Reed WM, Lee WB, Cawson JN, Brennan PC (2010) Malignancy Detection in Digital Mammograms. Academic Radiology 17: 1409–1413. 10.1016/j.acra.2010.06.016 20719545

[4] Rawashdeh M, Lee W, Pietrzyk M, Bourne R, Ryan E, Reed W, et al. (2013)The impact of using a JAFROC or ROC approach on the conclusions of a typical observer performance study; pp. 86730B-86730B-86736.

[5] Rawashdeh MA, Lee WB, Bourne RM, Ryan EA, Pietrzyk MW, Reed WM, et al (2013) Markers of Good Performance in Mammography Depend on Number of Annual Readings. Radiology 269: 61–67. 10.1148/radiol.13122581 23737538

[6] Bernardin HJ, Beatty RW (1984) Performance appraisal: Assessing human behavior at work Boston: Kent Pub. Co.

[7] Hunter JE (1983) A causal analysis of cognitive ability, job knowledge, job performance, and supervisor ratings In: Frank J. Landy SZ, Jeanette Cleveland, editor. Performance measurement and theory. Hillsdale, NJ: Lawrence Erlbaum Associates pp. 257–266.

[8] McCrae RR, Costa PT (1996) Toward a new generation of personality theories: Theoretical contexts for the five-factor model In: Wiggins JS, editor. The five factor model of personality: Theoretical perspectives. New York: Guilford Press pp. 51–87.

[9] Campbell JP, McCloy RA, Oppler SH, Sager CE (1993) A theory of performance In: Neal Schmitt WCB, editor. Personnel selection in organization. San Francisco: Jossey-Bass pp. 35–70.

[10] Campbell JP, Gasser MB, Oswald FL (1996) The substantive nature of job performance variability In: Murphy KR, editor. Individual differences and behavior in organizations. San Francisco: Jossey-Bass pp. 258–299.

[11] Hegarty M, Canham MS, Fabrikant SI (2010) Thinking about the weather: How display salience and knowledge affect performance in a graphic inference task. Journal of Experimental Psychology: Learning, Memory, and Cognition 36: 37–53. 10.1037/a0017683 20053043

[12] QuiŃOnes MA, Ford JK, Teachout MS (1995) The Relationship Between Work Experience And Job Performance: A Conceptual And Meta-Analytic Review. Personnel Psychology 48: 887–910.

[13] Balzer WK, Doherty ME, O'Connor R (1989) Effects of cognitive feedback on performance. Psychological Bulletin 106: 410–433.

[14] Waldie KE, Mosley JL (1996) Self-enhancing effect of social feedback on cognitive task performance. American Journal on Mental Retardation 100: 620–631. 8735575

[15] Motowildo SJ, Borman WC, Schmit MJ (1997) A Theory of Individual Differences in Task and Contextual Performance. Human Performance 10: 71–83.

[16] Pierce JL, Gardner DG, Cummings LL, Dunham RB (1989) Organization-Based Self-Esteem: Construct Definition, Measurement, And Validation. Academy of Management Journal 32: 622–648.

[17] Gardner DG, Pierce JL (1998) Self-Esteem and Self-Efficacy within the Organizational Context: An Empirical Examination. Group & Organization Management 23: 48–70.

[18] Sparrowe RT, Liden RC, Wayne SJ, Kraimer ML (2001) Social Networks and the Performance of Individuals and Groups. Academy of Management Journal 44: 316–325.

[19] Cross R, Cummings JN (2004) Tie and Network Correlates of Individual Performance in Knowledge-Intensive Work. Academy of Management Journal 47: 928–937.

[20] Burt RS (2000) The Network Structure Of Social Capital. Research in Organizational Behavior 22: 345–423.

[21] Burt RS (1992) Structural holes: The social structure of competition Cambridge, MA: Harvard university press.

[22] Marsden PV (1990) Network Data and Measurement. Annual Review of Sociology 16: 435–463.

[23] Wegener B (1991) Job Mobility and Social Ties: Social Resources, Prior Job, and Status Attainment. American Sociological Review 56: 60–71.

[24] Reagans R, Zuckerman EW (2001) Networks, diversity, and productivity: The social capital of corporate R&D teams. Organization Science: 502–517.

[25] Reagans R, McEvily B (2003) Network Structure and Knowledge Transfer: The Effects of Cohesion and Range. Administrative Science Quarterly 48: 240–267.

[26] Garton L, Haythornthwaite C, Wellman B (1997) Studying Online Social Networks. Journal of Computer-Mediated Communication 3: 0–0.

[27] Halgin DS, Borgatti SP (2012) An introduction to personal network analysis and Tie Churn statistics using E-NET. Connections 32: 37–48.

[28] Bdeir F (2013) Networks of inter-organisational coordination during disease outbreaks: University of Sydney.

[29] Coleman J, Katz E, Menzel H (1957) The Diffusion of an Innovation Among Physicians. Sociometry 20: 253–270.

[30] West E, Barron DN, Dowsett J, Newton JN (1999) Hierarchies and cliques in the social networks of health care professionals: implications for the design of dissemination strategies. Social Science & Medicine 48: 633–646. 10080364 10.1016/s0277-9536(98)00361-x

[31] Marsden PV (2002) Egocentric and sociocentric measures of network centrality. Social Networks 24: 407–422.

[32] Knoke D, Yang S (2008) Social Network Analysis. Thousand Oaks, CA: SAGE Publications, Inc.

[33] Meisel MK, Clifton AD, MacKillop J, Miller JD, Campbell WK, Goodie AS (2013) Egocentric Social Network Analysis of Pathological Gambling. Addiction (Abingdon, England) 108: 584–591. 10.1111/add.12014 PMC3578111 23072641

[34] Burt RS (1984) Network items and the general social survey. Social Networks 6: 293–339.

[35] Burt RS, Meltzer DO, Seid M, Borgert A, Chung JW, Colletti RB, et al (2012) What's in a name generator? Choosing the right name generators for social network surveys in healthcare quality and safety research. BMJ Quality & Safety 21: 992–1000. 10.1136/bmjqs-2011-000521 PMC12878522 22942400

[36] Chakraborty DP, Berbaum KS (2004) Observer studies involving detection and localization: Modeling, analysis, and validation. Medical Physics 31: 2313–2330. 15377098 10.1118/1.1769352

[37] Borgatti S (2006) E-NET software for the analysis of ego-network data Needham, MA: Analytic Technologies.

[38] Krackhardt D, Stern RN (1988) Informal Networks and Organizational Crises: An Experimental Simulation. Social Psychology Quarterly 51: 123–140.

[39] Teachman JD (1980) Analysis of Population Diversity: Measures of Qualitative Variation. Sociological Methods & Research 8: 341–362.

[40] Harrison DA, Klein KJ (2007) What's the difference? diversity constructs as separation, variety, or disparity in organizations. Academy of Management Review 32: 1199–1228.

[41] Suleiman WI, Lewis SJ, Georgian-Smith D, Evanoff MG, McEntee MF (2014) Number of mammography cases read per year is a strong predictor of sensitivity. Journal of Medical Imaging 1: 015503 10.1117/1.JMI.1.1.015503 26158030 PMC4478883

[42] Uzzi B, Spiro J (2005) Collaboration and Creativity: The Small World Problem. American Journal of Sociology 111: 447–504.

[43] P/L AsH (2008) The Role of the Radiologist in the Multidisciplinary Care Team. A Quality Use of Diagnostic Imaging Project. Final Report to the Royal Australian & New Zealand College of Radiologists. Sydney.

[44] Alcantara SB, Reed W, Willis K, Lee W, Brennan P, Lewis S (2014) Radiologist participation in multi-disciplinary teams in breast cancer improves reflective practice, decision making and isolation. European Journal of Cancer Care 23: 616–623. 10.1111/ecc.12169 24372588

[45] Van Deven T, Hibbert K, Faden L, Chhem RK (2013) The hidden curriculum in radiology residency programs: A path to isolation or integration? European Journal of Radiology 82: 883–887. 10.1016/j.ejrad.2012.12.001 23305755

